# High-Fat Diet-Related Obesity Promotes Urethane-Induced Lung Tumorigenesis in C57BL/6J Mice

**DOI:** 10.3389/fonc.2021.620993

**Published:** 2021-02-23

**Authors:** Dan Shi, Jingjing Wu, Youqile Wu, Xiaojing Lin, Cai Xu, Xuemei Lian

**Affiliations:** ^1^ Center for Lipid Research, Key Laboratory of Molecular Biology for Infectious Diseases, Ministry of Education, Chongqing Medical University, Chongqing, China; ^2^ Department of Nutrition and Food Hygiene, School of Public Health and Management, Chongqing Medical University, Chongqing, China; ^3^ Department of Child Health Care, Mianyang Maternity and Child Healthcare Hospital, Mianyang, China

**Keywords:** high fat diet, obesity, lung tumorigenesis, PI3K/Akt/mTOR, STAT3

## Abstract

Epidemiological studies have recently shown that obesity increases lung cancer risk, but the underlying biological connection is unclear. To determine whether high-fat diet (HFD)-induced obesity influences the susceptibility to chemical-induced lung tumorigenesis, a HFD feeding condition was combined with a multi-dose urethane-induced lung tumorigenesis model using C57BL/6J mice. In cell culture models, lung cancer cell lines A549 and H460 were used to investigate the effect of leptin on cell viability and its underlying mechanism of action. The results showed that obesity was induced with a 60 kcal% HFD feeding. Serum leptin levels increased with HFD feeding and further increased in urethane-administered and HFD-fed mice. Compared to the control diet-fed mice, the HFD-fed mice exhibited increased lung tumor burden and typical pro-tumorigenic STAT3 pathway activation in lung tissues after urethane administration. *In vitro*, leptin significantly increased the viability of lung cancer cell lines A549 and H460 in a dose-dependent manner by activation of STAT3, Bcl-2, and cyclin D1. These effects were significantly attenuated when PI3K or mTOR were inhibited by LY294002 or rapamycin, respectively. These results suggested that HFD-induced obesity could promote the development of lung tumorigenesis in C57BL/6J mice, and leptin-mediated activation of the PI3K/Akt/mTOR/STAT3 pathway was likely involved in this mechanism.

## Introduction

Lung cancer is the most common malignant tumor and the leading cause of cancer-related deaths worldwide. According to the GLOBOCAN data, the new lung cancer cases in 2018 globally were approximately 2.09 million, accounting for 11.6% of all new cases of cancer. Approximately 1.76 million deaths from lung cancer, accounting for 18.4% of all cancer deaths (1). Although smoking is the leading risk factor for lung cancer, there are other lifestyle-related risk factors involved as well.

With excessive energy intake and less physical activity, the world is in a fast-growing epidemic of obesity, especially in the developed countries. In 2014, the estimated age-standardized prevalence of obesity among men, women, and children was 10.8%, 14.9%, and 5.0%, respectively (2). Obesity can contribute to the morbidity and etiology of many diseases, including cardiovascular diseases, diabetes, steatohepatitis, and cancer, through chronic systematic inflammation and an unbalanced energy metabolism (3). A comprehensive systematic review conducted by the World Cancer Research Fund (WCRF) and the American Institute for Cancer Research (AICR) found that obesity was the risk factor for several cancer types, including postmenopausal breast cancer, colorectal cancer, and prostate cancer (4). In 2016, the International Agency for Research on Cancer (IARC)-working group reported that the risk of 13 types of cancer was positively related to obesity (2).

Although obesity has recently been identified as a major risk factor for pulmonary diseases, such as asthma, pulmonary hypertension, and acute respiratory distress syndrome (5), the relationship between obesity and lung cancer remains unclear. Numerous studies have reported that a higher body mass index (BMI) has an inverse relationship with lung cancer risk. However, the confounding effect of smoking or reverse causation due to preclinical weight loss cannot be disregarded (6). Recently, studies focusing on central obesity, namely abdominal obesity, have found that higher waist circumference (WC) and waist-to-hip ratio (WHR) are linked with increased lung cancer risk independent of BMI (7). Therefore, the relationship between obesity and lung cancer risk is not fully understood; hence, further studies are needed to elucidate the biological connection between obesity and lung tumorigenesis.

Leptin is a peptide hormone that is synthesized and secreted by white adipocytes and is highly expressed in obese individuals. Accumulating evidence suggests that leptin is involved in regulating tumor cell proliferation, invasion, and migration and is considered an intermediate for obesity-related cancers such as breast cancer and colon cancer (8). However, its role in lung tumorigenesis is not clear. In this study, obesity was induced by utilizing 60 kcal% high-fat feeding, and the role of leptin in lung tumorigenesis was elucidated by conducting *in vivo* and *in vitro* experiments. The results showed that high-fat diet (HFD)-related obesity could promote the development of urethane-induced lung cancer in C57BL/6J mice, and the leptin-mediated activation of PI3K/Akt/mTOR/STAT3 pathway was involved in the mechanism.

## Materials and Methods

### Animals and Experimental Design

Wild-type C57BL/6J male mice were purchased from and kept in the laboratory animal facility of Chongqing Medical University. All animal care and experimental procedures were reviewed and approved by the Animal Care and Use Committee at Chongqing Medical University (SYXK 2018-0003), and all procedures were performed in accordance with the relevant guidelines and regulations.

**Figure 1:**
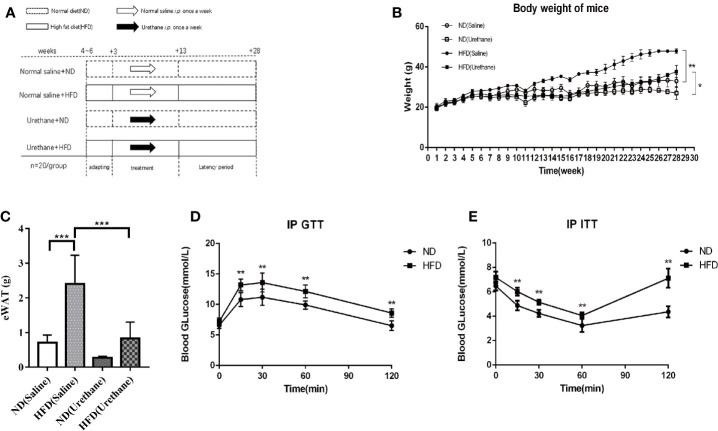
Differences in body weight, intraperitoneal glucose tolerance test (IP GTT) and insulin tolerance test (IP ITT) data among study groups. **(A)** Schematic diagram of the experimental design for multiple urethane injections induced lung carcinogenesis model in C57BL/6J mice. **(B)** Body weights of mice measured once a week. Dots indicate the weight mean per group. n=12-15 mice/group. **(C)** Epididymal WAT (eWAT) mass (n=4-5 mice/group). **(D)** For IP GTT, mice were made to fast overnight and injected with glucose (2 g/kg BW) intraperitoneally in the morning. Blood glucose levels were assessed using tail vein blood samples taken at baseline, 15, 30, 60, and 120 min after injection. n=10 mice/group **(E)** The IP ITT was performed in mice that were made to fast followed by intraperitoneal injection of insulin (0.75 U/kg BW) in 0.9% NaCl. Blood glucose levels were assessed using tail vein blood samples taken at baseline, 15, 30, 60, and 120 min after injection. n=10 mice/group. *P < 0.05, **P < 0.01, ***P < 0.001.

Six to eight-week-old male, wild-type C57BL/6J mice were purchased from the Experimental Animal Center of Chongqing Medical University and housed in the specified pathogen free (SPF) animal laboratory (constant temperature and humidity, 12 h light/dark cycle, and provided ad libitum access to sterile food and drinking water). Sixty mice were randomly divided into two groups: HFD (60 kcal% fat, D12492, Research Diets Inc., New Brunswick, NJ, USA) or isoenergetic control diet (ND, 10 kcal% fat, D12450B, Research Diets Inc.). The formula for the diets is presented in **Table 1**. After 3 weeks of adaptation to the feeding conditions, mice were further divided randomly into the experimental and control groups. All mice in the experimental group received an intraperitoneal injection of urethane (1 mg/g body weight, Sigma-Aldrich Inc., USA) once a week for 10 consecutive weeks, whereas the control group received the same volume of saline intraperitoneal injection. Lung tumorigenesis was assessed after 15-weeks latency period, as described in our previous study (**Figure 1A**) (9).

**Table 1:** Dietary composition and energy content of the experimental diets^a^.

Ingredient	ND	HFD
	g/kg	g/kg
Casein,80 Mesh	200	200
L-Cystine	3	3
Com Starch	315	0
Maltodextrin 10	35	125
Sucrose	350	68.8
Cellulose,BW200	50	50
Soybean Oil	25	25
Lard	20	245
Mineral Mix S10026	10	10
DiCalcium Phosphate	13	13
Calcium Carbonate	5.5	5.5
Phtassium Citrate,1 H2O	16.5	16.5
Vitamin Mix V10001	10	10
Choline Bitartrate	2	2
Energy, Kcal/g diet	3.85	5.24
%Energy Protein Carbohydrate Fat	/ 20 70 10	/ 20 20 60

a Prepared by Research Diets, Inc., New Brunswick, NJ. ND, control diet; HFD, high fat diet.

### Serum Collection

The mice were made to fast for 12 h before sample collection. Prior to euthanasia, 2% sodium pentobarbital was injected intraperitoneally at a concentration of 0.1 ml/10 g body weight. Then, cardiac blood was collected using a 1-ml syringe. After storage for 2–3 h at room temperature, the blood was centrifuged at 400 ×*g* for 20 min, and the serum was collected and stored at −80°C before use. Leptin expression in mouse serum was quantified using an ELISA development kit (USCN KIT Inc., Wuhan, China) according to the manufacturer’s recommendations.

### Intraperitoneal Glucose Tolerance Test (IP GTT) and Intraperitoneal Insulin Tolerance Test (IP ITT)

IP GTTs and IP ITTs were performed on mice fed different diets for 3 weeks. After fasting overnight, either glucose (2 g/kg of body weight) or insulin (0.75 U/kg of body weight in normal saline) was administered intraperitoneally to the mice, and blood glucose levels were assessed using the tail vein blood samples taken at baseline and at 15, 30, 60, and 120 min after injection.

### Bronchoalveolar Lavage Cell Counting

After the mice were euthanized, the trachea was exposed and a slow lavage with 1 ml of normal saline using an endotracheal tube was performed. Then, the lavage fluid was transferred to a pre-chilled EP tube. Bronchoalveolar lavage fluids (BALF) were centrifuged at 400 ×*g* for 10 min to separate the cells from the supernatants. Lavage cell counting was performed using a grid blood cell counting plate independently by three researchers.

### Lung Tumor Enumeration and Histology Assay

After the mice were euthanized, the trachea was exposed. With tracheal intubation, the lung tissue was expanded slowly by injecting approximately 3 ml of 4% paraformaldehyde. The surface lung tumors were carefully counted under a dissecting microscope by three experimental researchers who were blinded, and the nodule diameters were measured with the aid of digital calipers (10). The lungs were removed, fixed in paraformaldehyde for 24 h, dehydrated with gradient alcohol, embedded in paraffin, and sectioned into 5-μm-thick slices for hematoxylin & eosin (H&E) staining and immunohistochemistry (IHC) assay as described previously (9). Briefly, paraffin sections were obtained and deparaffinized. For H&E staining, slides were immersed in 100% alcohol for 5 min to drain xylene and then rehydrated with serial alcohol for 5 min each. The slides were stained in hematoxylin for 4–7 min, washed and put in 1% acid alcohol for 1–4 s, rinsed in running tap water and counterstained in Eosin Y for 3 min. For IHC assay, citrate buffer was used to retrieve the antigen. Afterwards, 3% H_2_O_2_-methanol solution was added at room temperature (15–25°C) for 15 min to block endogenous peroxidase activity, and 100–200 μl of ready-to-use goat serum was added and incubated for 30 min at room temperature. Next, the slices were incubated with antibodies against CD68 (1:100, BOSTER Biological Technology, China) or CD206 (1:250, Abcam, CA, United States) overnight at 4°C. The corresponding secondary antibodies were added and incubated at 37°C for 30 min. The DAB kit was used as a chromogenic agent and hematoxylin solution for counterstaining.

### Cell Culture

The adenocarcinoma human alveolar basal epithelial cell line A549 was purchased from the American Type Culture Collection (ATCC), and the human large cell lung cancer cell line NCI-H460 was purchased from the Cell Library of the Typical Culture Preservation Committee of the Chinese Academy of Sciences, Shanghai, China. Both cell lines were authenticated by Microread, Beijing, China. The A549 and H460 cell lines were maintained in Roswell Park Memorial Institute (RPMI)-1640 medium supplemented with 10% fetal bovine serum (Biological Industries, Israel) and 100 U/ml penicillin and streptomycin at 37°C in a 5% CO_2_ incubator.

Cell viability was assessed using the CCK-8 assay kit (MCE, USA). A549 or NCI-H460 cells were plated in 96-well plates at appropriate cell densities and cultured for 24 h in RPMI-1640 growth medium. After 16 h of starvation in serum-free medium, lung cancer cell lines were treated with recombinant human leptin (RD systems, USA) at increasing concentrations (0, 50, 100, and 200 ng/ml). At the indicated time point, 10 μl of CCK-8 solution was added to the wells, and the absorbance of each well was measured to calculate cell viability (%).

Cell cycle changes and apoptosis were detected using cell flow cytometry. Untreated A549 cells and cells treated with 100 ng/ml leptin were rinsed twice with PBS. The cells were then digested with trypsin, collected, and centrifuged at 400 ×*g* for 5 min. The cells for detecting cell cycles were fixed with 1 ml of ethanol for 24 h at 4°C, washed with 1× PBS, and incubated with 1 ml of propidium iodide (PI) for 30 min in the dark. DNA content was measured based on the presence of PI-stained cells. The cells used to detect apoptosis were pooled and subjected to Annexin V/PI staining using FITC Annexin V Apoptosis Detection kit I (BD Biosciences, USA) as per manufacturer’s instructions.

To determine the pathways associated with the effects of leptin on cell viability, A549 cells were incubated with 100 ng/ml rapamycin (Sangon Biotech, China) or 20 mmol/L LY294002 (Beyotime Biotechnology, China) alone or in combination with 100 ng/ml leptin (RD systems, USA) for 48 h.

### Western Blot Analysis

The lung tissues or treated A549 cells were harvested, and the whole proteins were extracted using a mixture of 100 µl RIPA lysate, 2 µl protease inhibitor (50×), and 1 µl 100 mM phenylmethanesulfonyl fluoride (PMSF, 100 mM). The protein concentration was determined using the bicinchoninic acid (BCA) method. Proteins were separated using sodium dodecyl sulfate polyacrylamide gel electrophoresis (Bio-Rad, Hercules, CA, USA) and transferred to PVDF membranes. Membranes were blocked in 5% non-fat milk in TBST for 1 h at 37°C and probed with primary antibodies overnight at 4°C. After three washes, the membranes were incubated with the corresponding secondary antibodies for 1 h at 37°C. Signals were detected with ECL western blot detection reagents (Millipore, Billerica, MA, USA) using a direct chemiluminescent detection system (Fusion 182Fx5, Vilber, France). Protein expression was quantitated using ImageJ software (NIH, Bethesda, MD, USA). The primary antibodies used were: mTOR (Cell Signaling Technology, USA), STAT3, Bcl-2 (BOSTER Biological Technology, China), cyclin D1 (Beyotime, Shanghai, China), β-actin (ZSGB-BIO, China), and GRP78 (a kind gift from Prof. Deqiang Wang, Chongqing Medical University, Chongqing, China).

### Statistical Analyses

The data are expressed as mean ± SEM. Differences between groups were compared using the Student’s *t*-test or two-way ANOVA with LSD post-hoc tests. The difference in tumor diameter distribution between the two diet groups was analyzed using the Chi-square test. Statistical analyses were performed using the Statistical Package for the Social Sciences Software Version 11.0 (SPSS, Chicago, IL, USA). P-value < 0.05 was considered as statistically significant.

## Results

### Effects of HFD on Body Weight, Glucose Tolerance, and Insulin Resistance in C57BL/6J Mice

The body weights of the mice in the experiment were measured once a week for 28 weeks. The average weight of mice and the epididymis adipose mass in the HFD group was significantly higher or larger than that of the isoenergetic control diet (ND) group (**Figures 1B, C**). Urethane administration induced body weight loss in all urethane administered mice (**Figure 1B**). The results of the IP GTT and IP ITT showed that the insulin resistance significantly increased in HFD-fed mice compared to that in ND-fed mice (**Figures 1D, E**). These data indicated that HFD induced obesity and related metabolic disturbances in this model.

### HFD Promotes Lung Tumorigenesis in Urethane-Administered Mice

**Figure 2:**
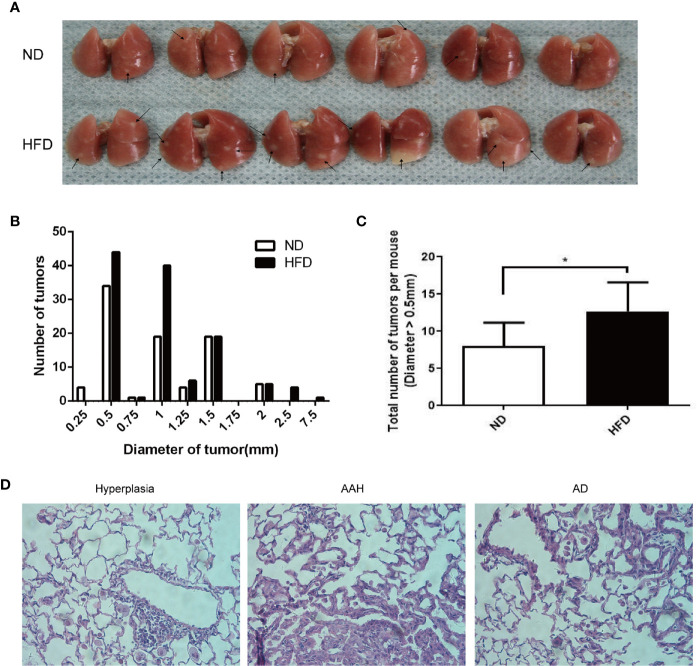
Increased burdens of lung tumor in HFD-fed C57BL/6J mice compared to that in ND-fed mice. **(A)** Gross photo of surface lung nodules in urethane-administered and HFD-fed and ND-fed mice. Arrows point to selective visible lung tumors. **(B)** Distribution of nodule diameters between two diet groups (n = 6 mice/group). **(C)** Numbers of nodule diameter larger than 0.5 mm between the two diet groups (n = 6 mice/group). *P < 0.05. **(D)** Representative H&E staining of lung sections in urethane-administered mice, showing epithelial cell hyperplasia (Left), atypical adenomatous hyperplasia (AAH, Middle) and papillary and solid adenoma (AD, right).

A lung cancer model in C57BL/6J mice using multiple urethane intraperitoneal injections was previously established in our lab and by other groups (9, 10). In this study, weekly intraperitoneal injection of urethane for 10 weeks caused a 100% lung tumor incidence (number of mice with tumors/number of mice injected) in both HFD-fed and ND-fed C57BL/6J mice (**Figure 2A**). Compared with the ND group, urethane-administered HFD-fed mice displayed an increasing trend in the numbers of large lung tumors (**Figure 2B**), and the numbers of tumor nodules larger than 0.5mm in diameter were increased significantly in HFD group (**Figure 2C**). Consistent with published data (9), atypical adenomatous hyperplasia (AAH) and papillary and solid adenoma (AD) were the two main neoplastic lesion types in this multi-dose urethane-induced lung cancer model (**Figure 2D**).

### HFD Enhances Lung Inflammation in Urethane-Administered Mice

**Figure 3:**
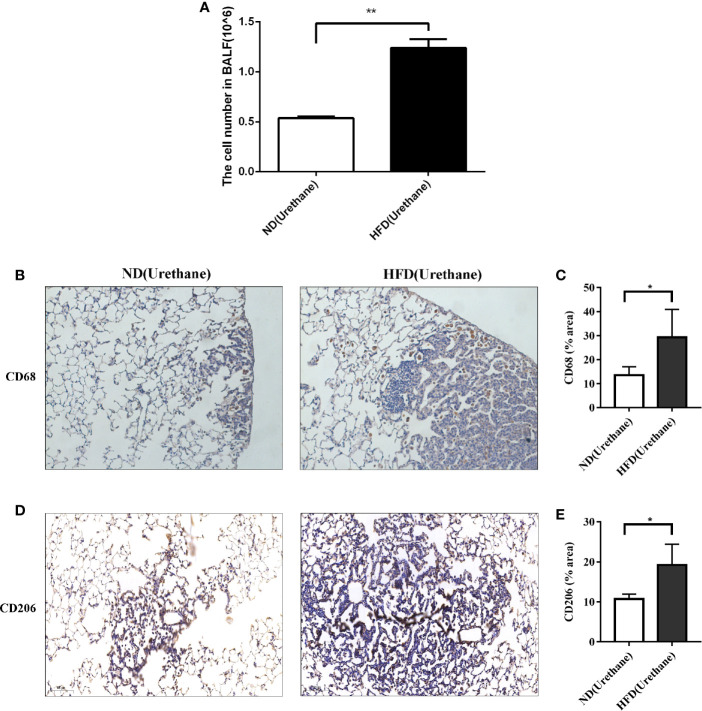
Infiltration of immune cells in bronchoalveolar lavage fluids and lung tissue. **(A)** Increased BALF cell count in urethane treated high-fat diet fed mice compared to urethane treated control diet fed mice (n=3 mice/group). **P < 0.01. **(B, C)** Increased expression of CD68 in lung tissue of urethane treated HFD-fed mice compared to urethane treated ND-fed mice. **(B)** Representative images of lung sections in urethane treated mice. Brown dots indicate the CD68 positive macrophages. (Original magnification: ×200). **(C)** Quantification analysis of immunohistochemistry related to CD68 between two diet groups by ImageJ. (n=4 mice/ group). *P < 0.05. **(D, E)** Increased expression of CD206 in lung tissue of urethane treated HFD-fed mice compared to urethane treated ND-fed mice. **(D)** Representative images of lung sections in urethane treated mice. Brown dots indicate the CD206 positive macrophages. (Original magnification: ×200). **(E)** Quantification analysis of immunohistochemistry related to CD206 between two diet groups by ImageJ. (n =4-5 mice/ group). *P < 0.05.

It has been reported that urethane-induced lung tumorigenesis is associated with severe inflammatory response (9). The results of BALF cell counting indicated that the total number of BALF cells was significantly higher in urethane-administered and HFD-fed mice than in the urethane-administered ND-fed mice (**Figure 3A**). Consistently, immunohistochemical results showed that increased CD68-positive macrophages appeared in the tumor site of HFD-fed mice than in that of ND-fed mice (**Figures 3B, C**), and majority of the macrophages presented a tumor associated M2 phenotype with positive CD206 staining (**Figures 3D, E**).

### HFD Promotes Leptin Expression in Urethane-Administered C57BL/6J Mice

**Figure 4:**
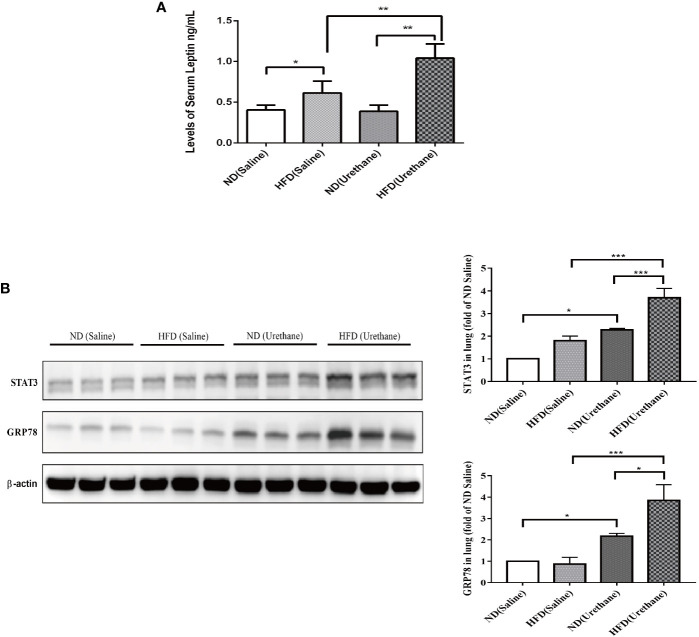
The expression of leptin in serum and target protein expression in lung tissues of C57BL/6J mice. **(A)** Serum leptin levels in different groups of C57BL/6J mice as measured by ELISA (n =3–5 mice/group). *P < 0.05, **P < 0.01. **(B)** Pulmonary expression of STAT3 and GRP78 in different groups of C57BL/6J mice shown by western blotting (n=3 mice/group). *P < 0.05, **P < 0.01, ***P < 0.001.

The serum leptin concentrations in HFD-fed mice were significantly higher than those in the ND-fed group, with the urethane-administered and HFD-fed mice having the highest level of serum leptin (**Figure 4A**). As a consequence, the typical leptin-regulated protein STAT3 and GRP78 expression levels significantly increased in lung tissues of urethane-administered and HFD-fed mice compared to those in urethane-administered ND-fed mice and all saline-administered mice, as shown using western blot (**Figure 4B**).

### Leptin Activates PI3K/mTOR/STAT3 Pathway to Promote Lung Cancer Cell Proliferation and Inhibit Lung Cancer Cell Apoptosis

**Figure 5:**
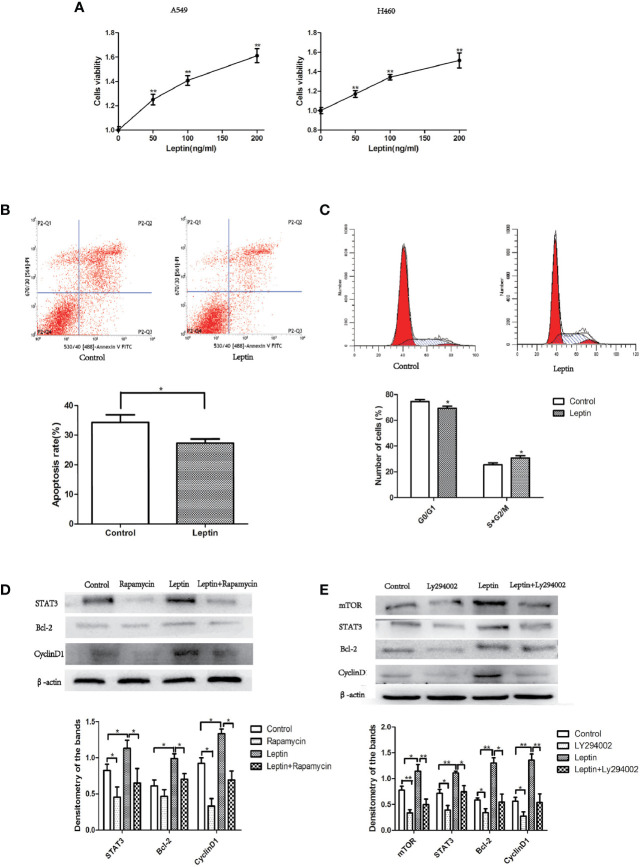
Effect of leptin on lung cancer cell proliferation and apoptosis *in vitro*. **(A)** Cell viability increased in a dose-dependent manner when A549 or H460 cells were treated with increasing concentrations of leptin (0, 50, 100, and 200 ng/ml). **(B)** Proportion of apoptotic cells decreased when A549 was treated with 100 ng/ml leptin as measured by flow cytometry assays. Upper: representative image of flow cytometry assay. **(C)** Cell cycle changes were observed when A549 was treated with 100 ng/ml leptin. Upper: representative image of flow cytometry assay. **(D)** Western blotting for the expression of STAT3, Bcl-2, and CyclinD1 in A549 lung cancer cells with or without the inhibition of mTOR. **(E)** Western blotting for the expression of mTOR, STAT3, Bcl-2, and CyclinD1 in A549 lung cancer cells with or without the Inhibition of PI3K. All quantifications are made from three individual experiments. *P < 0.05, **P < 0.01.

A549 and H460 lung cancer cells were treated with different concentrations of leptin for 48 h (0, 50, 100, and 200 ng/ml), and cell viability was determined using the CCK-8 assay. Compared to the control group (0 ng/ml), the cell viability of leptin-treated lung cancer cells increased significantly in a dose-dependent manner (**Figure 5A**). The flow cytometry results showed that the proportion of A549 cells in the S+G2/M phase significantly increased after treatment with leptin (100 ng/ml) for 48 h (**Figure 5B**). In addition, the apoptosis rate of A549 cells treated with leptin (100 ng/ml) for 48 h was significantly lower than that of the control group (0 ng/ml) (**Figure 5C**). Consistently, the cell cycle-and apoptosis-related protein expressions of mTOR, STAT3, Bcl-2, and cyclin D1 significantly increased in A549 lung cancer cells after treatment with 100 ng/ml leptin. In the presence of LY294002 (PI3K inhibitor) or rapamycin (mTOR inhibitor), leptin-induced activation of STAT3, Bcl-2, and cyclin D1 was significantly attenuated (**Figures 5D, E**).

## Discussion

Lung cancer is not among the well-defined obesity-related cancers. Although the enhancing effect of HFD on 4-nitroquinoline 1-oxide-induced pulmonary tumorigenesis in ICR male mice was reported as early as in 1989 (11), the underlying mechanisms related to obesity and lung cancer risk have not been investigated in animal models largely because the majority of studies published indicated that high BMI had an inverse relationship with lung cancer risk in humans (6). Recently, central obesity was reported to heighten the lung cancer risk in multiple epidemiological studies and meta-analyses (7, 12), which prompted our interest in further understanding the potential biological connection. In this study, we investigated whether HFD-induced obesity augmented the risk of urethane-induced lung cancer in C57BL/6J mice, and the results showed that HFD did promote cancer growth in the urethane-induced lung cancer model. Elevated levels of serum leptin were observed, and the classical pro-tumorigenic STAT3 pathway was activated in lung tissues of HFD-fed and urethane-administered mice. Furthermore, leptin could stimulate the proliferation of lung cancer cells in a PI3K/Akt/mTOR-dependent manner *in vitro*.

HFD feeding (typically 60% energy from animal fat) is a commonly used method to generate an obesity mouse model. In this study, HFD feeding resulted increased weight gain and larger epididymis adipose mass compared to ND-fed mice (**Figures 1B, C**). Even though urethane administration caused a decrease in body weight, the body weight of the urethane-administered and HFD-fed mice was significantly higher than that of the urethane-administered ND-fed mice (**Figure 1B**). Additionally, IP GTT and IP ITT showed disturbed glucose metabolism in HFD-fed mice (**Figures 1D, E**).

Urethane-induced mouse lung cancer model is well established in lung tumorigenesis studies (13). Our previous study found that multi-dose injection of urethane for a period of 10 weeks and continuous feeding for 15 weeks could lead to 100% incidence of lung tumors in C57BL/6J mice (9). Using this urethane-induced lung cancer model, we observed that the lung tumor burdens (especially nodule volumes) were significantly higher in HFD-fed mice than in ND-fed mice (**Figures 2B, C**). Bartsch et al. suggested that chronic inflammation was associated with many human cancers (14), and inflammation also played an important role in urethane-induced lung cancer in C57BL/6J mice (10). We observed increased BALF cell numbers and pulmonary CD68-positive macrophage infiltration in urethane-administered and HFD-fed mice compared to those in ND-fed mice (**Figure 3**), consistent with the increased lung cancer burden in urethane-administered and HFD-fed mice.

Leptin is the first adipocyte-derived cytokine (adipokine), and its level increase in adipose tissue associated with obesity correlates with increases in serum leptin levels. In addition, leptin plays a critical role in regulating appetite and energy balance, and it has been shown to activate cell proliferation and survival in cancer cells, including those of the prostate, breast, endometrium, and colon (15). Leptin acts *via* its receptor (LepRb), while LepRb possesses no intrinsic tyrosine kinase activity. Janus family tyrosine kinases (JAKs) are receptor-associated protein tyrosine kinases, which are utilized by LepRb to phosphorylate the receptor itself as well as targets such as signal transducers and activators of transcription proteins (STATs). Therefore, the JAK/STAT signaling pathway plays a critical role in mediating the effects of leptin and further induces its own gene expression (16). Strong evidence has demonstrated that aberrant STAT3 signaling promotes the initiation and progression of human cancers (including lung cancer) by either inhibiting apoptosis or inducing cell proliferation, angiogenesis, invasion, and metastasis (17). Consistent with the increased serum leptin level, the protein expression of STAT3 was elevated in the lung tissues of HFD-fed and urethane-administered mice compared to that in ND-fed urethane-administered mice (**Figures 4A, B**).

Endoplasmic reticulum (ER) stress is a hallmark of obesity. The most important ER retention chaperone glucose-regulated protein 78 (GRP78) has an important role in cancer (18). Leptin can upregulate GRP78 expression through the PI3K/TOR/STAT3 signaling pathway in neuronal and lung cancer cells (19, 20). An elevated pulmonary GRP78 expression was also observed in the lung tissues of HFD-fed and urethane-administered mice (**Figure 4B**).

The effect and potential mechanism of leptin on lung cancer cell growth was further demonstrated *in vitro*. We showed that leptin significantly increased the viability of lung cancer cells A549/H460 in a dose-dependent manner (**Figure 5A**). Leptin-treated A549 cells triggered more cells to enter the S+G2/M phase (**Figure 5B**) and decreased the apoptotic rate compared to that in the control group (**Figure 5C**). Leptin knockdown was previously found to inhibit cell proliferation and induce apoptosis of NSCLC cell lines (21). The mammalian target of rapamycin (mTOR) is a key molecule regulating metabolism and cell growth. Dysregulation of the PI3K/Akt/mTOR pathway is involved in the development of non-small cell lung cancer (NSCLC) (22). For the first time, we found that the PI3K/Akt/mTOR/STAT3 pathway was also involved in the effect of leptin on lung cancer cell growth (**Figures 5D, E**).

In summary, the role of HFD-induced obesity on lung tumorigenesis was elucidated both *in vivo* and *in vitro* in this study, and highly expressed leptin mediated the activation of STAT3 pathway was likely involved in the mechanism.

With obesity and metabolism regulation becoming an emerging frontier in lung physiopathological research, this study provided a preliminary biological connection between obesity and lung cancer and might facilitate further studies into the relationship between obesity and lung cancer risk. The leptin deficient ob/ob mouse model is a better choice to confirm the exact role of leptin. In addition, the effect of other obesity-related signaling molecules on lung cancer development such as insulin, insulin-like growth factor-1, adiponectin, steroid hormones, and cytokines needs to be elucidated. More rigorously designed and uniform cohort studies are needed to confirm the relationship between obesity and lung cancer risk in the future.

## Data Availability Statement

The original contributions presented in the study are included in the article/supplementary material. Further inquiries can be directed to the corresponding author.

## Ethics Statement

The animal study was reviewed and approved by Animal Care and Use Committee at Chongqing Medical University.

## Author Contributions

DS, JW, XJL, and CX conducted the experiments. JW and YW analyzed the data, DS and YW wrote the manuscript. XML designed the research and revised the manuscript. All authors contributed to the article and approved the submitted version.

## Funding

This work was supported by the National Natural Science Foundation of China (No.81071907, 81973030) and the Project of Technology Innovation and Application Development of Chongqing (cstc2019jscx-msxmX0280).

## Conflict of Interest

The authors declare that the research was conducted in the absence of any commercial or financial relationships that could be construed as a potential conflict of interest.

## Abbreviations

AAH, Atypical adenomatous hyperplasia; AICR, American Institute for Cancer Research; ATCC, American Type Culture Collection; BMI, Body mass index; ER, Endoplasmic reticulum; H&E, Hematoxylin & eosin; HFD, High-fat diet; NSCLC, Non-small cell lung cancer; PI, Propidium iodide; SPF, Specified pathogen free; STAT, Signal transducers and activators of transcription; WC, Waist circumference; WCRF, World Cancer Research Fund; WHR, Waist-to-hip ratio.
